# CARING FOR PARENTS AND IN-LAWS IN CHINA: IMPLICATIONS FOR COGNITIVE FUNCTIONING OF ADULT CHILD CAREGIVERS

**DOI:** 10.1093/geroni/igac059.2543

**Published:** 2022-12-20

**Authors:** Zhiyong Lin, Haoshu Duan

**Affiliations:** University of Texas at San Antonio, San Antonio, Texas, United States; University of Maryland, Baltimore, Maryland, United States

## Abstract

Considerable research and public discourse on family caregiving portrays it as a stressful and burdensome experience with serious negative health consequences. Yet, there is also recent evidence indicating better health status in caregivers compared to non-caregivers. Especially for cognitive health, caring for others involves cognitively stimulating activities that can help prevent cognitive decline. Although the negative consequences of eldercare on the mental and physical health of caregivers are well-documented, how it would affect their cognitive functioning is underexplored. Using three waves of nationally representative data in China, the China Health and Retirement Longitudinal Study (CHARLS, 2011, 2013, 2018), this study investigated the longitudinal association between parental caregiving and cognitive functioning among adult child caregivers, and further examined how this association was conditioned on the relationship type (parent versus in-law), caregiving frequency, and gender of adult children. Descriptive analysis indicated that women are more likely to be occasional caregivers for their own parents and regular caregivers for parents-in-law compared to men. Results from multilevel mixed-effects models showed that caring for own parents was beneficial for adult children’s cognitive functioning whereas caring for parents-in-law was not significantly associated with their cognitive functioning. Both sons and daughters benefited from caring for their own parents although the beneficial effect was stronger for sons. Thus, we recommend to consider relationship types between caregivers and care-recipients when investigating informal caregiving and various health outcomes. Furthermore, we suggest more policies and programs that aim to help children-in-law who look after their parents-in-law.

